# Neurosurgical Management of Interspinous Device Complications: A Case Series

**DOI:** 10.3389/fsurg.2022.841134

**Published:** 2022-03-16

**Authors:** T. J. Florence, Irene Say, Kunal S. Patel, Ansley Unterberger, Azim Laiwalla, Andrew C. Vivas, Daniel C. Lu

**Affiliations:** ^1^UCLA Department of Neurosurgery, Los Angeles, CA, United States; ^2^Department of Neurosurgery, University of Massachusetts, Worcester, MA, United States; ^3^David Geffen School of Medicine, University of California, Los Angeles, Los Angeles, CA, United States

**Keywords:** lumbar stenosis, interspinous device, decompressive laminectomy, minimally invasive (MIS), complications

## Abstract

**Background:**

Best practice guidelines for treating lumbar stenosis include a multidisciplinary approach, ranging from conservative management with physical therapy, medication, and epidural steroid injections to surgical decompression with or without instrumentation. Marketed as an outpatient alternative to a traditional lumbar decompression, interspinous process devices (IPDs) have gained popularity as a minimally invasive stabilization procedure. IPDs have been embraced by non-surgical providers, including physiatrists and anesthesia interventional pain specialists. In the interest of patient safety, it is imperative to formally profile its safety and identify its role in the treatment paradigm for lumbar stenosis.

**Case Description:**

We carried out a retrospective review at our institution of neurosurgical consultations for patients with hardware complications following the interspinous device placement procedure. Eight cases within a 3-year period were identified, and patient characteristics and management are illustrated. The series describes the migration of hardware, spinous process fracture, and worsening post-procedural back pain.

**Conclusions:**

IPD placement carries procedural risk and requires a careful pre-operative evaluation of patient imaging and surgical candidacy. We recommend neurosurgical consultation and supervision for higher-risk IPD cases.

## Introduction

Degenerative lumbar stenosis is a condition resulting from severe narrowing of the spinal canal and often manifests as neurogenic claudication: back and/or leg pain exacerbated by load-bearing activity and lumbar extension, and improved symptoms with rest or flexion. Standard of care treatment begins with conservative measures such as physical therapy, and anti-inflammatory pain medications. Treatment escalates stepwise to corticosteroid injections and decompressive surgery with or without instrumentation for refractory symptoms and corresponding radiographic pathology. Developed as an alternative to decompressive laminectomy, interspinous process devices (IPDs) are an emerging technology in treating lumbar stenosis. The devices are designed to limit the extension between two spinal levels, in turn preventing symptomatic exacerbation of lumbar stenosis. Chiefly placed by interventional pain specialists or physiatrists according to 2018 CMS data, patient selection and IPD placement are performed by physicians without dedicated training in spine instrumentation (1).

Several IPD brands are available, including X-STOP (Medtronic, Minneapolis, MN), Coflex (Paradigm Spine, New York, NY), Helifix (Alphatec, Carlsbad, CA), Stenofix (Depuy Synthes, Raynham, MA), FLEXUS (Globus, Audubon, PA), Device for Intervertebral Assisted Motion (DIAM) (Medtronic, Minneapolis, MN), Aperius (Medtronic, Minneapolis, MN), Wallis (Zimmer Biomet, Warsaw, IN), and the Superion (Vertiflex/Boston Scientific, Marlborough, MA) (2–4). Efficacy studies have shown an improvement in back and leg pain, functional outcome scores, and reduced the opioid medication requirement compared to conservative therapy (5–11). However, the optimal role of IPDs relative to surgical decompression remains unclear (12, 13). Heterogeneity in practice patterns reflects a lack of clear clinical evidence for the role of IPDs in the management of lumbar stenosis. We frequently observe device implantation offered without a formal evaluation from a spine surgeon.

In this study, we describe our case series of patients referred to our service for management of complications after undergoing placement of IPD by non-surgical providers. We detail a novel surgical approach for minimally invasive IPD removal and simultaneous definitive decompression. We measured parameters describing stenosis and spinal alignment and then discussed each case as a representative example of an area of concern with IPDs.

## Materials and Methods

Institutional review board approval was obtained for this study. A database of neurosurgery consultations was reviewed to identify inpatient and outpatient consultations regarding issues with previously placed IPDs. Electronic charts were queried for patient presentation, imaging findings, management decision-making, and short- and long-term outcomes. In cases requiring surgical intervention, intraoperative video footage was collected.

We extracted spinal parameters from available clinical images, covering time points before IPD implantation, post-implantation at the time of neurosurgical evaluation, and post-evaluation images. Within-patient measurements were performed on identical imaging modalities where possible. To minimize errors associated with cross-modality comparisons (e.g., MRI to CT) between patients, we utilized ratiometric measurements. To evaluate stenosis, we define relative canal diameter as the dorsal-ventral canal lumen diameter at the maximally stenotic symptomatic level, divided by the diameter at the immediately rostral pedicle. This measurement borrows from established quantitative methods (14) for measuring stenosis with the added numerical benefit of normalizing for individual anatomy. We define lumbar lordosis as the Cobb angle formed by the L1 and S1 superior vertebral body endplate on standing, neutral-position lumbar radiographs, in keeping with established methods (15, 16). Across patients, we calculate means for defined time points and test for significance *via* Student's *t*-test.

## Summary of Cases

**Figure 1:**
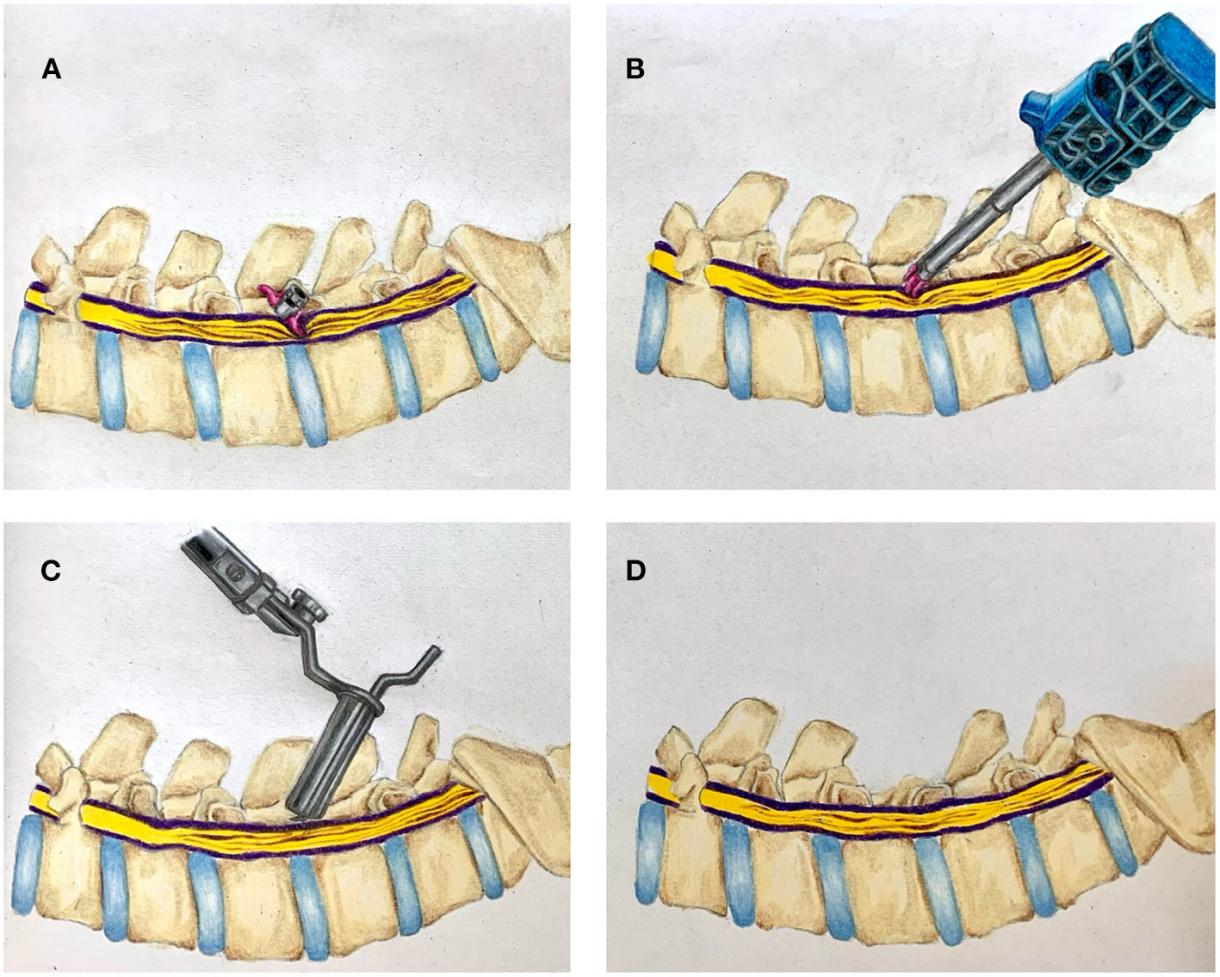
Illustration of combined interspinous process device (IPD) retrieval and MIS lumbar decompression. **(A)** Migrated interspinous process device *in situ*. **(B)** Retrieval of migrated IPD. **(C)** Tubular MIS laminectomy. **(D)** Completed laminectomy.

Cases are summarized in brief in Tables 1–3. Cases 1–4 describe inpatient consultations; cases 5–8 describe outpatient consultations. A graphical illustration of our minimally invasive surgery (MIS) method for IPD removal and simultaneous definitive laminectomy is shown in Figure 1.

**Table 1:** Patient demographics.

**Patient**	**Age**	**Sex**	**Comorbidities**	**Presenting Sx**	**Presenting pathology**	**Initial pain regimen**	**ESI**	**PT**	**Preop nsg consult**
1	75	M	Locally invasive prostate CA	cLBP, Neurogenic Claudication, BLE L5 Radiculopathy	Severe L4/5 stenosis	Percocet, gabapentin	No	Yes	No
2	84	M	CAD s/p CABG, HFrEF, AfIb, pHTN, CVA	cLBP	Severe L4/5 stenosis	Oxycodone	Yes	Yes	No
3	58	M	Afib, poorly controlled T2DM	cLBP	Baastrup's disease, spondylosis without canal stenosis	Meloxicam, flexeril, gabapentin	Yes	Yes	No
4	91	F	CAD s/p CABG, pHTN, COPD	R L5 radiculopathy	Moderate L4/5 and L5/S1 stenosis, RL5 synovial cyst w/severe foraminal stenosis	Norco and pregabalin	Yes	Yes	Yes
5	78	M	HCM, pAfib	Neurogenic claudication	Moderate L3/4 and L4/5 stenosis	Norco	Yes	Yes	No
6	73	F	Osteoporosis, HCV	cLBP, BLE L5 radiculopathy	Severe L4/5 stenosis, degenerative levoscoliosis	Meloxicam, robaxin, nortriptyline	Yes	Yes	No
7	77	F	None	L5 radiculopathy	Severe L3/4 and L4/5 stenosis	Ibuprofen	Yes	No	No
8	74	F	RA, coronary aneurysm, pHTN, COPD, emphysema	Rheumatic joint pain, BLE L5 radiculopathy	Severe L4/5 stenosis	Tramadol, meloxicam, gabapentin, duloxetine	Yes	Yes	No

*Afib, atrial fibrillation; BLE, bilateral lower extremity; CA, cancer; CAD, coronary artery disease; CABG, coronary artery bypass graft; cLBP, chronic low back pain; CVA, cerebrovascular accident (stroke); ESI, epidural steroid injections; HCM, hypertrophic cardiomyopathy; HCV, hepatitis C; nsg, neurosurgery; pHTN, pulmonary hypertension; PT, physical therapy; Sx, symptoms; T2DM, type 2 diabetes mellitus*.

**Table 2:** Perioperative considerations.

**Patient**	**Off-label**	**Implant level**	**Complication**	**Prompting Sx**	**Surgery**	**Outcome**
1	Yes	L4/5	Ventral migration	Immediate post-operative pain exacerbation	MIS L4/5 laminectomy, IPD removal	Pain exacerbation resolved
2	Yes	L4/5	L4 spinous process fracture, ventral migration	Acute LBP, L4/5 radiculopathy	Bone fragment and IPD removal (performed by pain team)	BLE L4 radiculopathy
3	Yes	L3/4	None	Acute pain exacerbation	None	Requires frequent RFA ablations
4	Yes	L4/5	Inferior and ventral migration, S1 stenosis	Extreme BLE L5/S1 radiculopathy urinary retention	MIS L4/5 laminectomy IPD removal	Resolved radiculopathy and urinary retention
5	No	L3/4 and L4/5	None	Progressive R L4/5 radiculopathy	L3/4 4/5 laminectomy IPD removal x 2	Radiculopathy resolved
6	Yes	L4/5	None	Neurogenic claudication, worsening BLE L5 radiculopathy	MIS L4/5 laminectomy, IPD removal	R thigh pain resolved, L persistent
7	Yes	L3/4 and L4/5	None	Nonrelief of symptoms	L3/4 4/5 laminectomy IPD removal x 2	Resolved radiculopathy
8	Yes	L4/5	None	Nonrelief of symptoms	MIS L4/5 laminectomy, IPD removal	Resolved radiculopathy

*BLE, bilateral lower extremity; IPD, interspinous process device; LBP, low back pain; MIS, minimally invasive surgery; RFA, radiofrequency ablation; Sx, symptoms*.

**Table 3:** Symptomatology and temporal characteristics.

**Patient**	**Pain at consultation** **(VAS)**	**Pain at follow up** **(VAS)**	**Implant to consultation** **(days)**	**Implant to surgical intervention** **(days)**	**Follow-up** **(days)**
1	10/10	8/10	3	4	894
2	6/10	8/10	11	21	211
3	7/10	7/10	17	n/a	949
4	10/10	2/10	7	9	378
5	8/10	3/10	874	905	68
6	8/10	6/10	266	290	147
7	10/10	4/10	115	173	330
8	8/10	8/10	359	383	108

*VAS, visual analog scale*.

### Case 1

**Figure 2:**
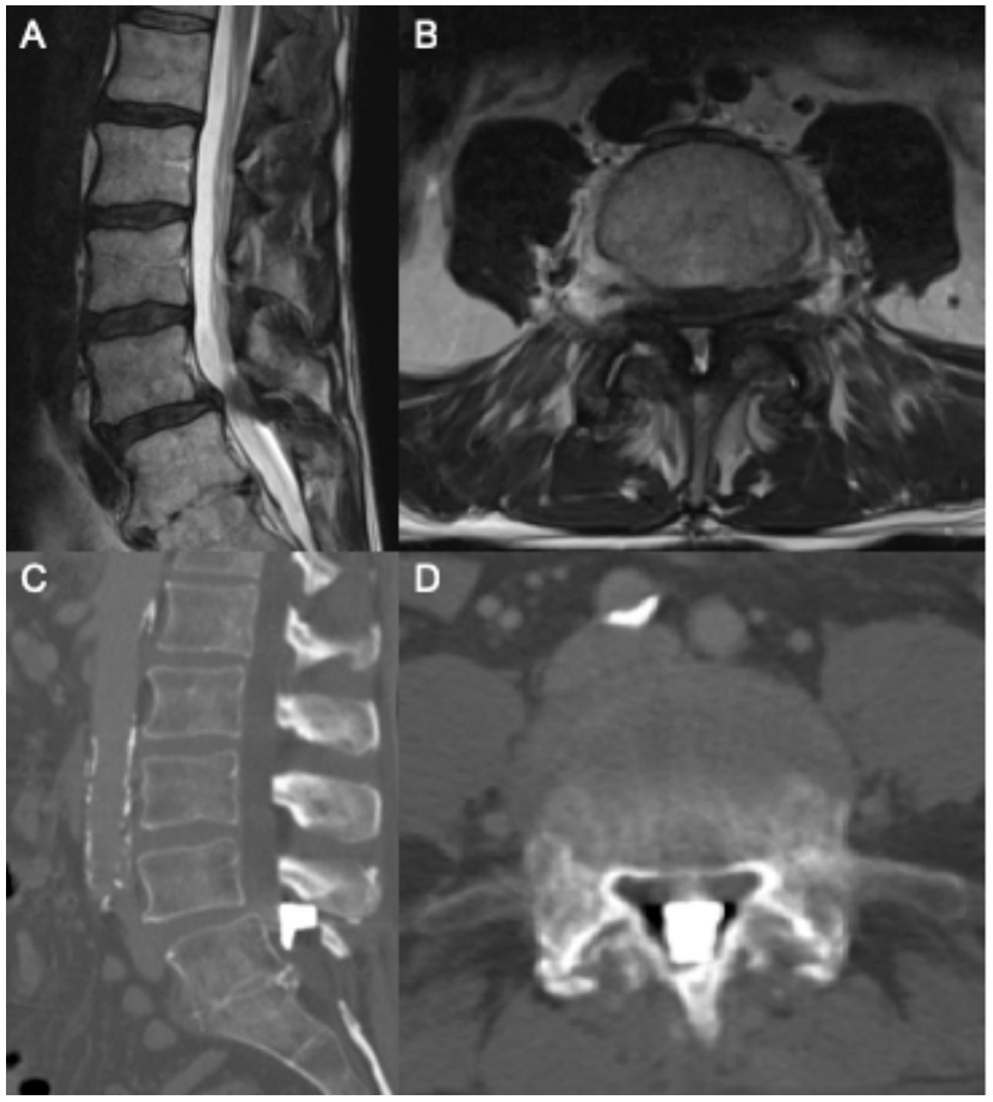
Pre-operative sagittal **(A)** and axial **(B)** T2 MRI showing L4/5 severe central canal stenosis. Following interspinous spacer placement, sagittal **(C)** and axial **(D)** CT scan showing spacer migration into central canal.

A 75-year-old man presented with neurogenic claudication for 2 months. MRI showed lumbar spondylosis with severe lumbar stenosis at the L4/5 level (Figures 2A,B). He was seen by a pain management physician with an initial trial of conservative management including physical therapy and anti-inflammatory medications. He continued to have severe pain with disability. He had no neurologic weakness, sensory changes, or bowel and/or bladder dysfunction. At this time, he was recommended an interspinous spacer placement and had the Boston Scientific Superion interspinous spacer placed by an outside physician at the L4/5 level.

The patient was evaluated at our institution after this procedure with worsening severe back pain. He did not have a neurologic deficit, or bowel/bladder dysfunction. A CT scan of the lumbar spine was ordered and showed the interspinous spacer device had migrated anterior to the L4/5 interspinous space, leading to further central canal stenosis (Figures 2C,D). Neurosurgery was consulted for recommendations on management for migration of the interspinous spacer device. Given the patient's worsening symptoms and imaging findings, the patient was taken to the operating room within 24 h of presentation to remove the device.

The patient was positioned prone, and the previous incision was located and opened. Subperiosteal dissection was completed to identify the L4 and L5 spinous processes. Soft tissue was removed in the interspinous space until the dorsal side of the interspinous spacer device was identified. The device had migrated anteriorly to the lamina. A laminotomy at L4 was completed to retrieve the device. The dura was examined after removal of the device with no evidence of a cerebrospinal fluid leak. A decompression at L4/5 was completed, given the patient's degenerative lumbar stenosis with identified hypertrophied facet joints and thickened ligaments (Supplementary Video 1).

Post-operatively the patient's back pain and neurogenic claudication were significantly improved. The patient was discharged on post-operative day 1 with oral pain medications. There were no long-term issues with pain or neurologic function.

### Case 2

**Figure 3:**
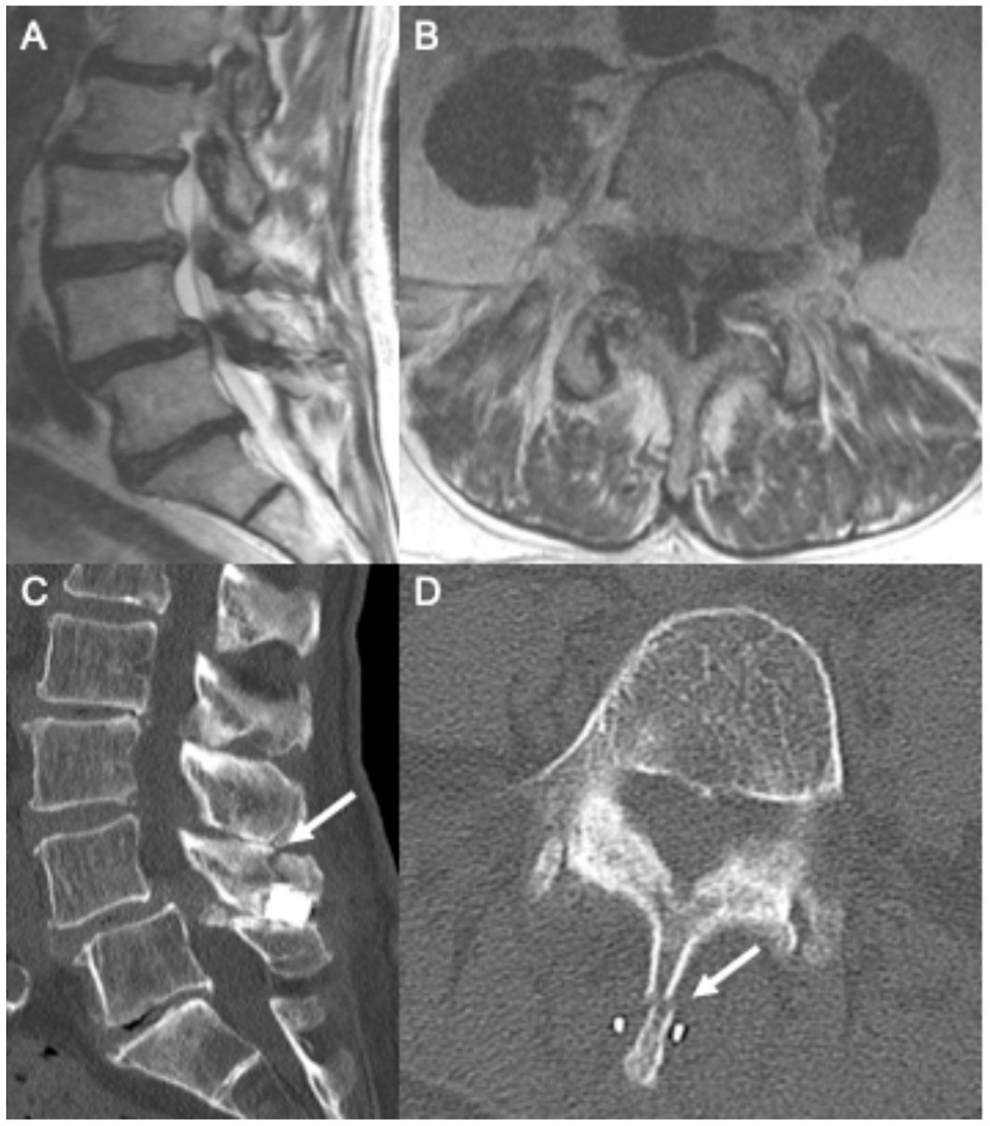
Pre-operative sagittal **(A)** and axial **(B)** T2 MRI showing grade 1 spondylolisthesis and L4/5 severe central canal stenosis. Following interspinous spacer placement, sagittal **(C)** and axial **(D)** CT imaging showing L4 spinous process fracture.

An 84-year-old gentleman with coronary artery disease with recent placement of drug-eluting stents and congestive heart failure with an ejection fraction of 20% presented with chronic back pain, neurogenic claudication, and right-sided radicular pain in the L5 distribution. The patient had no weakness or bowel and bladder dysfunction. The patient's MRI showed severe lumbar stenosis at the L4/5 level and mild stenosis at the L3/4 level (Figures 3A,B). He was followed by a pain management team outside our department who recommended IPD placement after finding no relief with conservative measures. The patient's anticoagulation was held for the procedure, and a Superion interspinous spacer device was implanted at the L4/5 level.

The patient presented to our emergency department 1 week after this procedure with worsening back pain and no improvement in pre-operative radicular leg pain and paresthesia. There was no change in strength or bowel/bladder function. A plain X-ray in the emergency department showed a L4 spinous process fracture (Figures 3C,D). At this time, neurosurgery was consulted. Removal of the IPD was recommended because of new worsening back pain and instability of the IPD.

The patient was positioned prone, and the previous incision was opened. The interspinous spacer device was removed along with the fracture fragment of the L4 spinous process. Post-operatively, the patient's pain improved, and the patient was discharged on the same day of the procedure.

### Case 3

A 58-year-old man with atrial fibrillation initially presented with chronic low back pain without neurogenic claudication or radicular pain. He was initially managed by an outside clinical team who diagnosed L4/5 Baastrup's disease and performed a partial removal of the L4 spinous process and lamina. After this procedure, the patient had persistent lower back pain. The patient had no neurologic weakness or bowel and/or bladder symptoms. An MRI showed lumbar spondylosis without significant central canal stenosis. A Superion interspinous spacer device was placed at the L3/4 level. His anticoagulation was held for this procedure.

The patient presented to our emergency department of our institution with worsening pain over the incision site used to place the interspinous spacer device. Neurosurgery was consulted for recommendations on management. Imaging completed in the emergency department showed no fracture or migration of the device. The patient's pain was able to be controlled with pain medications, and he was scheduled for facet injections at this level.

### Case 4

A 91-year-old female with coronary artery disease status post three-vessel bypass, pulmonary hypertension, and chronic obstructive pulmonary disease presented with debilitating right lower extremity radiculopathy. Imaging revealed a synovial cyst at the right L5/S1 facet resulting in severe foraminal stenosis and moderate L4/5 and L5/S1 canal stenosis. She was evaluated by both neurosurgical and orthopedic specialists who did not recommend surgical intervention given her age and serious comorbidities. She established care with a pain specialist who first managed her conservatively with oral pain medication and epidural and foraminal steroid injections. Ultimately, she underwent implantation of an L4/5 IPD by an interventional pain specialist.

Upon awakening in the recovery unit, the patient developed severe surgical site pain and new bilateral lower extremity radiculopathy. She required admission for the pain control. Our neurosurgical service was consulted after several days of unremitting pain and urinary retention. CT lumbar spine revealed ventral migration of the IPD into the canal with severe stenosis. The patient underwent an urgent MIS L4/5 laminectomy and IPD removal with subsequent resolution of pain and urinary retention. Due to deconditioning, the patient was discharged to a skilled nursing facility.

### Case 5

A 78-year-old male with hypertrophic cardiomyopathy and paroxysmal atrial fibrillation presented with chronic low back pain and neurogenic claudication. After no response to physical therapy, oral pain medications, and epidural steroid injections, he underwent implantation of IPD at L3/4 and L4/5 for moderate stenosis by an interventional pain specialist.

The patient presented to our clinic with persistent back pain and new right L5 radiculopathy. Workup revealed subtle progression of stenosis, including the right L4 lateral recess. We performed a minimally invasive removal of both IPDs and simultaneous L3/4 and L4/5 laminectomy with a partial right L4 medial facetectomy. The patient's right L5 radiculopathy and neurogenic claudication symptoms were resolved.

### Case 6

A 73-year-old female with osteoporosis, hepatitis C, and lumbar spondylosis presented with symptomatic severe stenosis at L4/5 and mild degenerative levoscoliosis. She suffered from debilitating low back pain and severe bilateral radiculopathy in an L5 distribution. These symptoms were managed conservatively by an interventional pain specialist with NSAIDs, antidepressants, muscle relaxants in addition to steroid injections and physical therapy. Eventually this provider implanted an IPD at the L4/5 level.

Following implantation, the patient experienced worsening bilateral L5 radiculopathy. She sought outpatient neurosurgical consultation at our institution. We performed an MIS removal of the IPD with simultaneous decompression of L4/5. She had complete resolution of right thigh pain and significant improvement in her left thigh pain.

### Case 7

A 77-year-old female with no significant past medical history presented with severe bilateral L5 radiculopathy. Imaging demonstrated severe lumbar stenosis at L3/4 and L4/5. Her pain became refractory to epidural steroid injections, and an outpatient pain specialist implanted IPDs at L3/4 and L4/5.

After 6 months of persistent symptoms, the patient presented for outpatient neurosurgical consultation. Imaging demonstrated bilateral nerve root compression, and we removed the IPD and performed laminectomies at L3/4 and L4/5. On outpatient follow-up, the patient's bilateral radicular leg symptoms were resolved.

### Case 8

A 74-year-old female with rheumatoid arthritis, coronary aneurysm, pulmonary hypertension, chronic obstructive pulmonary disease, and emphysema presented to our outpatient clinic for neurosurgical consultation. She suffered from chronic low back pain, neurogenic claudication, and bilateral L5 radiculopathy. A previous MRI demonstrated severe stenosis at L4/5. Her symptoms had been managed by an outpatient pain specialist with oral pain medication, antidepressants, physical therapy, and steroid injections. One year prior to the presentation, she underwent L4/5 IPD placement at an out-of-state medical center.

On presentation to our spine clinic, she had experienced no significant improvement in any of her symptoms referable to lumbar stenosis in the intervening year. Repeat lumbar X-ray demonstrated an L4/5 IPD in stable position; a new MRI redemonstrated severe L4/5 stenosis without significant progression. We therefore felt the IPD had failed to address the symptomatic stenosis and that the patient would benefit from surgical decompression. Simultaneous IPD removal and MIS laminectomy were performed *via* the technique described above. At follow-up the patient reported a significant reduction in back pain, claudication symptoms, and radiculopathy.

## Results

For each patient, we examined the timing of surgical consultation and its effect. Mean time from the IPD placement to the neurosurgical consultation was 206 days (SD 301 days); mean time from the IPD placement to the surgical intervention was 255 days (SD 322 days). Mean follow-up duration for patients in this series was 386 days (SD 347 days). Visual analog scale (VAS) pain scores decreased from a mean of 8.4 on initial consultation to 5.6 at last follow-up.

**Figure 4:**
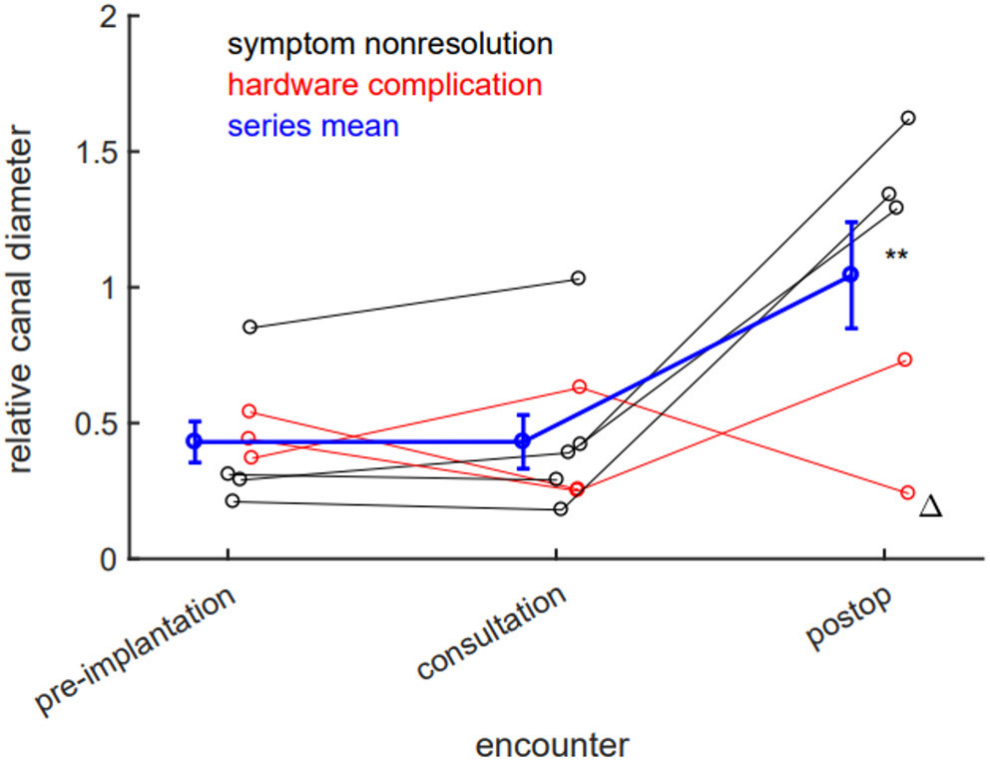
Laminectomy, but not interspinous process device (IPD) implantation, reduces lumbar stenosis. There is no significant radiographic evidence of canal stenosis reduction between implantation and neurosurgical consultation. Canal stenosis only improves in a statistically significant manner after laminectomy. Black datapoints represent patients seen for symptom nonresolution; red datapoints represent patients seen for hardware complications; blue data represent population mean; error bars ±SEM. **p < 0.05 (0.02, post-op compared to either pre-implantation or consultation stenosis). Delta (Δ) denotes patient whose IPD was explanted by interventional pain team.

We sought to systematically study imaging parameters better to understand the effects of IPD placement and its removal. First, we examined the effect of IPD placement on lumbar canal stenosis. We define a dimensionless measure, “relative canal diameter,” as the dorsal-ventral canal lumen diameter at the maximally stenotic symptomatic level, divided by the diameter at the immediately rostral pedicle. We found no measurable improvement in canal stenosis from IPD placement at the time of neurosurgical consultation (pre-implantation 0.430, consultation 0.431, *p* = 0.99). Statistically significant improvement in canal stenosis in our case series was observed only after definitive surgical decompression (post-op 1.044, *p* = 0.02 when compared both with pre-implantation and consultation stenosis; Figure 4).

**Figure 5:**
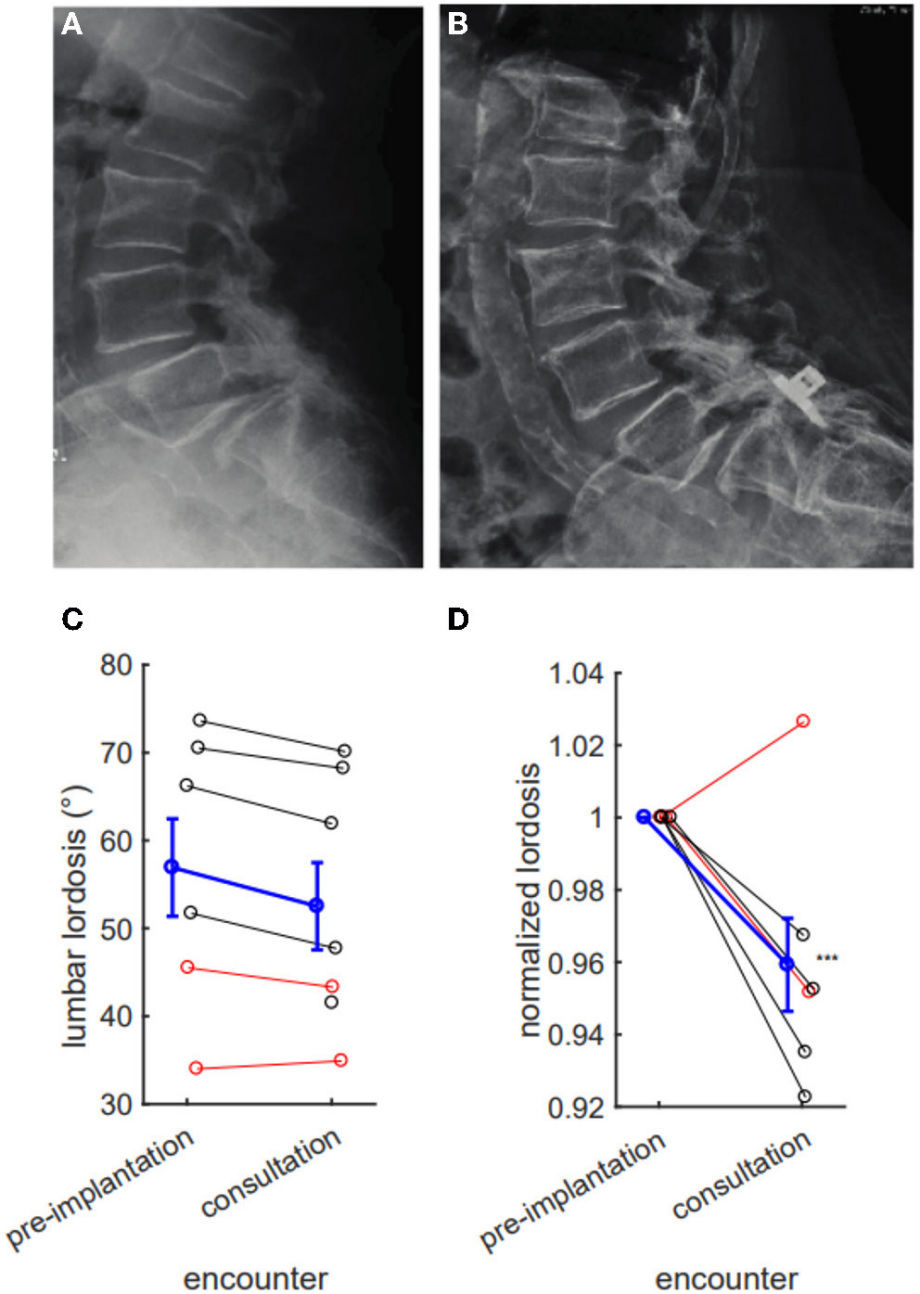
Interspinous process device (IPD) implantation results in a measurable reduction in lumbar lordosis. Pre- **(A)** and post- **(B)** placement X-rays with ventral migration of device into L5/S1 interspace, with evident reduction in lumbar lordosis. **(C)** IPD implantation tends to reduce lumbar lordosis (pre-implantation mean 56.92 degrees, consultation 52.51 degrees, *p* = 0.60), but this difference fails to reach statistical significance. **(D)** This reduction reaches significance when normalizing for pre-implantation lordosis (consultation 95.9% of baseline, difference 4.1%, *p* = 0.0075). **(C,D)** Red denotes hardware complications; black denotes nonresolution of symptoms; blue denotes series mean. Error bars mean ± SEM ***p < 0.01.

We next examined if IPD implantation affects spinal alignment. Specifically, we hypothesized that implantation might reduce lumbar lordosis by holding two lumbar levels in relative flexion. Across all eight patients, we did not observe a statistically significant absolute reduction in lumbar lordosis, likely due to intrinsic variability (pre-implantation mean 56.92 degrees, consultation mean 52.51 degrees, *p* = 0.60). When this variability was controlled by baseline normalization, we observed a significant 4.1% relative reduction in lumbar lordosis after IPD implantation (*p* = 0.0075; Figure 5).

## Discussion

Interspinous process devices were placed without a formal neurosurgical consultation in all, except one case. While patient stated upon interview that he was not initially interested in surgery, the other patients answered that they would have considered surgery as a treatment option. This patient cohort skews elderly with multiple severe medical comorbidities. We surmise that prior treating physicians may have assumed that these patients were not candidates for surgery, discouraging referrals. We wish to emphasize that the final assessment of surgical candidacy is a joint risk–benefit analysis between the operating surgeon, the patient, anesthesiologist, and consultant physicians for perioperative risk stratification. In this cohort, we observed a delay in definitive treatment, associated with a delay in neurosurgical consultation. Furthermore, we were not aware that any of these patients were assessed pre-operative to their index surgery for risk assessment and optimization for anesthesia.

Furthermore, there is a logical contradiction in deeming a patient not a surgical candidate for one procedure while recommending another. This practice pattern arises from the assumption that IPD placement is significantly less invasive than a laminectomy and could be performed under conscious sedation. On the contrary, published data suggest that minimally invasive lumbar decompression compares favorably to interspinous device implantation in terms of operative time, estimated blood loss, and recovery (17); additionally, MIS procedures, including advanced instrumentation procedures such as transforaminal lumber interbody fusion (TLIF), are now performed routinely under conscious sedation (18). Therefore, we expect differences in perioperative risks to be minimal (19). Minimally invasive decompressive surgery is well established as a short, safe procedure with high satisfaction rates (20). There is no data demonstrating reduced perioperative morbidity with IPD placement vs. surgical decompression.

In fact, recent research on IPD has been largely promising, with several studies reporting long-term, cost-effective benefit in large cohorts (7–10). Registry data of high patient satisfaction, decreased opioid consumption, and even randomized controlled trials support its use (6, 21–23). However, the majority of these studies were industry sponsored. While industry partnerships remain integral to technological innovation, it is clear that further objective study is needed.

Our study observed a high rate of ventral and intracanalicular hardware migration, which all risk permanent nerve injury, leading to weakness, bowel/bladder dysfunction—all device-related complications beyond the purview of physiatry and pain medicine. Spinal instrumentation failure and misplacement fall well outside their scope of practice, and several interventional pain specialists have recognized their shortcomings in surgical training (22, 24, 25). Yet, the CPT code for the IPD placement, 22,869 is frequently billed by non-surgical spine providers as a “stabilization/distraction device,” and a recent investigation suggests that its lucrative fee scheduling may influence practice patterns (26). In the interest of patient safety and full transparency, we emphasize a neurosurgical spine consultation prior to IPD placement.

Furthermore, we observed a seemingly arbitrary, unsubstantiated expansion of indications for IPD placement beyond what is supported by clinical data. Outcome analysis spanning up to 5 years after implantation concluded that patients with moderate lumbar stenosis are the best candidates for IPD (7, 8). However, 75% (6 out of 8) patients in our series demonstrated severe lumbar stenosis. Patient 4 in our case series is particularly illustrative. Her radiculopathy stemmed from a synovial cyst causing foraminal stenosis. Rather than undergoing a foraminal decompression, she was recommended for IPD placement by an interventional pain specialist. Spinal instrumentation requires a nuanced, comprehensive understanding of biomechanics and pathophysiology, and recognition of these subtleties hold real-world consequences for patients.

Despite its minimally invasive deployment, IPD is hardware instrumentation of the lumbar spine. We show that distraction of posterior spinal elements from an IPD reduces lumbar lordosis. Despite its minimally invasive deployment, IPD is hardware instrumentation of the lumbar spine. We show distraction of posterior spinal elements from an IPD reduces lumbar lordosis. We observe a roughly 4% reduction in lumbar lordosis across patients in this series. This is a relatively large change surprisingly uncompensated by increased lordosis at other lumbar levels. The clinical significance of this change is indeterminate; our sample is biased to include only patients with post-implantation complications. Furthermore, it is not clear if this change is transient or permanent. A simple hypothesis is that patients with device-associated pain exaggerate lumbar flexion away from instrumented levels. To evaluate this hypothesis, lumbar lordosis should be measured in cohorts with and without post-placement complications. The data do, however, highlight the ability of IPD placement to alter sagittal parameters.

Interventional pain medicine provides physicians with robust procedural exposure, ranging from image-guided injections, ablations, and blocks, but implantation of spinal instrumentation represents an unprecedented foray into spine surgery. What is most concerning is not simply the procedure itself, but the absence of careful consideration of and deliberation on spinal biomechanics. Spinal instrumentation is typically placed by fellowship-trained orthopedic and neurological surgeons with several years of advanced education, careful apprenticeship, and supervised surgical training in spine pathology. Pain specialists performing IPD implantation simply lack formal training in basic surgical technique, let alone minimally invasive spine surgery.

In August 2021, the AANS–CNS Joint Section on Disorders of the Spine and Peripheral Nerves released a position statement on spinal instrumentation by non-surgeon spine practitioners (27). Naming IPD devices specifically, the document cites concerns about the lack of standardized, formal training in pathology recognition and treatment formulation, inability to address potential complications, and unintentional alterations in spinal balance parameters and biomechanics. In our case series, we document patient examples of each of these areas of concern. Multidisciplinary collaboration with pain specialists and physiatrists is essential. However, we remain firm in our conclusion that spinal instrumentation, however minimally invasive, should be performed by fellowship-trained spine surgeons.

Complications of IPD placement have been explored previously in patient series large and small (28–38). The present study is novel in several ways. First, we are the first to measure alterations in sagittal parameters and lumbar stenosis as a function of IPD placement and MIS decompression. Second, although dorsal device migration and spinous process fracture have been previously reported, ventral device migration into the lumbar central canal has not. We report two such cases resulting in severe iatrogenic in the short series presented here. For both patient counseling and expert consultation, awareness of the totality of device complications is critical.

The major limitation of our study is that it is an uncontrolled case series, constituting a low level of clinical evidence. Furthermore, our case series of referred patients are biased toward complication and treatment failure. Yet, our series supports that (1) IPD is subject to hardware complications and treatment failure and (2) spine consultation should be sought before placement. Given the proliferation of IPD devices, we firmly believe in spreading awareness and promoting patient safety for all spine patients and the neurosurgical community.

## Conclusions

In this study, we illustrate eight cases of patient complications after IPD placement. We describe hardware migration, hardware-related fracture, and a lack of post-procedural improvement. Therefore, we recommend consultation with a fellowship-trained spine surgeon for any patient considering IPD placement.

## Data Availability Statement

The raw data supporting the conclusions of this article will be made available by the authors, without undue reservation.

## Ethics Statement

The studies involving human participants were reviewed and approved by UCLA IRB. Written informed consent for participation was not required for this study in accordance with the national legislation and the institutional requirements. Written informed consent was not obtained from the individual(s) for the publication of any potentially identifiable images or data included in this article.

## Author Contributions

TF conceived study, performed data analysis, and authored manuscript. IS authored manuscript. KP conceived study, authored manuscript, and edited operative video. AU provided illustration. AV contributed data and edited manuscript. DCL conceived the study, guided data analysis, and authored manuscript. All authors contributed to the article and approved the submitted version.

## Conflict of Interest

The authors declare that the research was conducted in the absence of any commercial or financial relationships that could be construed as a potential conflict of interest.

## Publisher's Note

All claims expressed in this article are solely those of the authors and do not necessarily represent those of their affiliated organizations, or those of the publisher, the editors and the reviewers. Any product that may be evaluated in this article, or claim that may be made by its manufacturer, is not guaranteed or endorsed by the publisher.

## Supplementary Material

Supplementary Video 1 Video recording of removal of interspinous device that has migrated into the central canal. The previous incision was opened, and monopolar electrocautery was used to expose the dorsal surface of the device between the L4 and L5 spinous processes. A high-speed drill and Kerrison Rongeurs were used to remove part of the L4 and L5 spinous processes to free the interspinous spacer device. A tool designed to place the device is used in reverse (in this case, the Boston Scientific Superion “Inserter”). Briefly, the insertion tool is docked onto the dorsal aspect of the implant. A drive screw mechanism in the handle of the insertion tool is used to retract the IPD interspinous blades. The device was removed en bloc, and a microsurgical lumbar decompression was completed.
